# Serum Fibroblast Growth Factor 21 Levels Are Positively Associated with Metabolic Syndrome in Patients with Type 2 Diabetes

**DOI:** 10.1155/2019/5163245

**Published:** 2019-09-10

**Authors:** Ruo-Yao Gao, Bang-Gee Hsu, Du-An Wu, Jia-Sian Hou, Ming-Chun Chen

**Affiliations:** ^1^School of Medicine, Tzu Chi University, Hualien, Taiwan; ^2^Division of Nephrology, Hualien Tzu Chi Hospital, Buddhist Tzu Chi Medical Foundation, Hualien, Taiwan; ^3^Division of Metabolism and Endocrinology, Hualien Tzu Chi Hospital, Buddhist Tzu Chi Medical Foundation, Hualien, Taiwan; ^4^Department of Pediatrics, Hualien Tzu Chi Hospital, Buddhist Tzu Chi Medical Foundation, Hualien, Taiwan

## Abstract

**Background:**

Fibroblast growth factor 21 (FGF21) acts as a potent metabolic regulator. Serum FGF21 levels were significantly higher in obesity and type 2 diabetes mellitus (T2DM) populations. The aim of this study was to evaluate the relationship between serum FGF21 levels and metabolic syndrome (MetS) in T2DM patients.

**Methods:**

Fasting blood samples were obtained from 126 T2DM patients. MetS and its components were defined according to the diagnostic criteria from the International Diabetes Federation. Serum FGF21 concentrations were measured using a commercially available enzyme-linked immunosorbent assay.

**Results:**

Among these patients, 84 (66.7%) had MetS. Female gender, hypertension, systolic blood pressure (SBP), diastolic blood pressure (DBP), waist circumference (WC), body weight (BW), body mass index (BMI), body fat mass, fasting glucose, glycated hemoglobin level (HbA1c), triglyceride level (TG), urine albumin-to-creatinine ratio (UACR), insulin level, homeostasis model assessment of insulin resistance (HOMA-IR), and FGF21 levels were higher, whereas high-density lipoprotein cholesterol level (HDL-C) and estimated glomerular filtration rate (eGFR) were lower in DM patients with MetS. Univariate linear analysis revealed that hypertension, BMI, WC, body fat mass, SBP, DBP, logarithmically transformed TG (log-TG), low-density lipoprotein cholesterol (LDL-C) level, log-glucose, log-creatinine, log-UACR, log-insulin, and log-HOMA-IR positively correlated, whereas HDL-C and eGFR negatively correlated with serum FGF21 levels in T2DM patients. Multivariate forward stepwise linear regression analysis revealed that body fat mass (adjusted *R*^2^ change = 0.218; *P*=0.008) and log-TG (adjusted *R*^2^ change = 0.036; *P* < 0.001) positively correlated, whereas eGFR (adjusted *R*^2^ change = 0.033; *P*=0.013) negatively correlated with serum FGF21 levels in T2DM patients.

**Conclusions:**

This study showed that higher serum FGF21 levels were positively associated with MetS in T2DM patients and significantly positively related to body fat mass and TG but negatively related to eGFR in these subjects.

## 1. Introduction

Type 2 diabetes mellitus (T2DM), a chronic metabolic disease characterized by hyperglycemia and insulin resistance, is a significant health problem and global burden, with an increasing prevalence worldwide [1]. According to the data from the International Diabetes Federation, 336 million people were diagnosed with T2DM globally in 2011, and the figure is expected to elevate to 552 million by 2030 [2]. Metabolic syndrome (MetS), with a prevalence rate of 23.6% among adults in the European country according to the National Cholesterol Education Program Adult Treatment Panel III definition, is an independent risk factor for T2DM and cardiovascular disease (CVD) [2, 3]. The MetS population is expected to have a two- to five-fold risk of developing DM and heart disease over the following 5–10 years than people without MetS [4].

Fibroblast growth factor 21 (FGF21) is a polypeptide with 210 amino acids from a human gene located on chromosome 19 at the 5′ region of the 1,2-fucosyltransferase. It is produced preferentially in the liver [5] and has been identified as an endocrine and metabolic hormone because of its potent effect on lipid and glucose metabolism and on insulin sensitivity and energy balance [6]. An animal study revealed that FGF21 had favorable effects of lowering serum glucose and triglyceride (TG) levels and improving lipoprotein profiles in genetic compromised FGF transgenic mice and primates [7, 8]. However, pieces of evidence of FGF21 as a potential disease marker for human metabolic-related illness are growing. Epidemiology studies revealed that higher serum FGF21 is an independent predictor of the MetS in Asian individuals and FGF21 levels elevated significantly among prediabetic and diabetic patients and can predict the diabetes development in a Chinese population [9, 10]. Eto et al. and Bobbert et al. also represented that the circulating FGF21 concentrations have a positive association with parameters in T2DM Japanese patients and the occurrence of MetS and T2DM in Caucasian patients, respectively [11, 12]. Taken together, FGF21, from physiological and clinical perspectives, is a potential biomarker for the early detection of human metabolic disorder.

Although emerging studies have evaluated the relationships of this hepatokine to obesity-related disease, whether or not FGF21 predicts MetS in T2DM patients, the interrelationships of FGF21 with the metabolic parameters among these populations have not been described in detail. Therefore, we investigated how circulating FGF21 levels are correlated with metabolic parameters in T2DM Taiwanese patients with MetS.

## 2. Materials and Methods

### 2.1. Participants

This study was approved by the Protection of the Human Subjects Institutional Review Board of Tzu Chi University and Hospital and was conducted in accordance with the Declaration of Helsinki. Diabetes mellitus (DM) was diagnosed as the fasting plasma glucose was either ≥126 mg/dL or if the 2 h glucose during an oral glucose tolerance test was ≥200 mg/dL or using oral hypoglycemic medications or insulin [13]. Written informed consent was obtained from all participants prior to enrolling in this study. Finally, from November 2014 to March 2015, a total of 126 patients with T2DM follow-up in the metabolic outpatient department at Buddhist Tzu Chi General Hospital, Hualien, Taiwan were enrolled. After the participant was seated for at least 10 min, blood pressure (BP) was measured in the morning using standard mercury sphygmomanometers with appropriate cuff sizes. Systolic BP (SBP) and diastolic BP (DBP) were taken three times at 5 min intervals and were averaged for analysis. Hypertensive patients were diagnosed based on SBP ≥140 mmHg and/or DBP ≥90 mmHg or taking any antihypertensive medication in the past 2 weeks. If patients had an acute infection, heart failure, and malignancy at the time of blood sampling, or if they refused to provide informed consent for the study, they were excluded.

### 2.2. Anthropometric Analysis

In light clothing and without shoes, the body weight (BW) and body height of each participant were measured to the nearest 0.5 kg and 0.5 cm, respectively. With the hands on the hips, waist circumference (WC) was assessed using a tape around the waist from the point between the lowest ribs and the hip bones. Body mass index (BMI) was calculated as weight in kilograms divided by height in square meters. Bioimpedance measurements of body fat mass were performed at the bedside according to the standard tetrapolar whole body (hand-foot) technique using a single-frequency (50 kHz) analyzer (Biodynamic-450; Biodynamics Corporation, Seattle, USA). All measurements were performed by the same operator [14–16].

### 2.3. Biochemical Investigations

Following an overnight fast, approximately 5 mL blood samples of all participants were immediately centrifuged at 3000 g for 10 min. Serum concentrations for blood urea nitrogen (BUN), creatinine, fasting glucose, glycated hemoglobin (HbA1c), TG, total cholesterol, high-density lipoprotein cholesterol (HDL-C), and low-density lipoprotein cholesterol (LDL-C) were measured using an autoanalyzer (Siemens Advia 1800; Siemens Healthcare GmbH, Henkestr, Germany) [14–16]. Urine albumin-to-creatinine ratio (UACR) was measured using a random spot urine test. Serum FGF21 (Phoenix Pharmaceuticals, Inc. Burlingame, CA, USA) concentrations were measured using commercially available enzyme immunoassay kits, and serum insulin (Labor Diagnostika Nord, Nordhorn, Germany) concentrations were determined using a commercially available enzyme-linked immunosorbent assay [14–16]. Insulin resistance was evaluated using homeostasis model assessment of insulin resistance (HOMA-IR) as follows: HOMA-IR = fasting serum insulin (*μ*U/mL) × fasting plasma glucose (mg/dL)/405 [14–16]. The estimated glomerular filtration rate (eGFR) was calculated using the Chronic Kidney Disease Epidemiology Collaboration equation in this study.

### 2.4. MetS and Its Components

The prevalence of MetS was defined according to the International Diabetes Federation definition [17]. People had central obesity with a WC ≥90 cm in men or ≥80 cm in women (Chinese criteria) and matched two or more of the following criteria: fasting serum glucose ≥100 mg/dL, TGs ≥150 mg/dL, HDL-C level <40 mg/dL in men or <50 mg/dL in women, or BP ≥130/85 mmHg were classified as having MetS. The use of antihypertensive drugs was considered as high BP in this analysis. T2DM was determined using the World Health Organization criteria [13]. A patient was considered as having DM if the fasting plasma glucose was ≥126 mg/dL or if he or she was undergoing an antidiabetic therapy.

### 2.5. Statistical Analysis

Data were tested for normal distribution using the Kolmogorov–Smirnov test. Normally distributed data were expressed as mean ± standard deviation, and comparisons between patients were performed using the Student's independent *t*-test (two-tailed). Data not normally distributed were expressed as medians and interquartile ranges, and comparisons between patients were performed using the Mann–Whitney *U* test (TG, fasting glucose, HbA1c, BUN, creatinine, insulin, HOMA-IR, and FGF21). Data expressed as the number of patients were analyzed by the *χ*^2^ test. FGF21 levels were tested for independency associated with MetS by the multivariate logistic regression analysis. Because TG, fasting glucose, HbA1c, BUN, creatinine, insulin, HOMA-IR, and FGF21 levels were not normally distributed, they underwent base 10 logarithmic transformations to achieve normality. Clinical variables that correlated with serum FGF21 levels in patients with T2DM were evaluated using univariate linear regression analysis. Variables that were significantly associated with FGF21 levels in patients with T2DM were tested for independency by multivariate forward stepwise regression analysis. Data were analyzed using SPSS for Windows (version 19.0; SPSS Inc., Chicago, IL, USA). A *P* value < 0.05 was considered as statistically significant.

## 3. Results

Table 1 shows the laboratory and clinical characteristics of the 126 enrolled T2DM patients. A total of 84 patients (66.7%) had MetS. Patients who had MetS had significantly higher serum FGF21 levels than those without MetS (*P* < 0.001). Compared with DM patients without MetS, those with MetS showed a much higher proportion of women (*P*=0.008) and as expected more hypertension (*P* < 0.001); higher SBP (*P* < 0.001) and DBP (*P* < 0.001); higher WC (*P* < 0.001); higher BW (*P* < 0.001), BMI (*P* < 0.001), and body fat mass (*P* < 0.001); higher fasting glucose (*P*=0.004), HbA1c level (*P*=0.005), UACR (*P* < 0.001), TG (*P* < 0.001), insulin level (*P* < 0.001), and HOMA-IR (*P* < 0.001); and lower HDL-C concentrations (*P*=0.003) and eGFR (*P*=0.003). No statistically significant differences in MetS were found in terms of use of statins, fibrates, or antidiabetic drugs.

The unadjusted and multivariate logistic regression analysis of FGF21 levels with other factors associated with MetS is presented in Table 2. The unadjusted serum FGF21 levels with MetS showed that FGF21 increased per 1 pg/mL (odds ratio (OR): 1.008, 95% CI: 1.003–1.012, *P*=0.001) increased the 0.8% risk of MetS in patients with T2DM. Multivariate logistic regression analysis adjusted for age and gender revealed a 0.7% increase in the risk of MetS (adjusted OR 1.007, 95% CI: 1.002–1.011, *P*=0.004) for every 1 pg/mL increase in FGF21 (Model 1). After multivariate logistic regression analysis with Model 1 added with eGFR and UACR, an increased 0.5% risk of the MetS (adjusted OR 1.005, 95% CI 1.001–1.010, *P*=0.027) was observed for every 1 pg/mL increase in FGF21 (Model 2). Multivariate logistic regression analysis using Model 2 with added serum insulin level and HOMA-IR also revealed an increased 0.5% risk of MetS (adjusted OR 1.005, 95% CI: 1.000–1.010, *P*=0.035) for every 1 pg/mL increase in FGF21 (Model 3). Each of these analyses confirmed that serum FGF21 level is positively associated with MetS in patients with T2DM.

The univariate and multivariate linear regression analyses of the clinical variables associated with fasting serum FGF21 levels in patients with T2DM are presented in Table 3. Hypertension (*r* = 0.201, *P*=0.024), BMI (*r* = 0.259, *P*=0.003), WC (*r* = 0.301, *P*=0.001), body fat mass (*r* = 0.359, *P* < 0.001), SBP (*r* = 0.191, *P*=0.032), DBP (*r* = 0.180, *P*=0.043), logarithmically transformed TG (log-TG; *r* = 0.499, *P* < 0.001), LDL-C level (*r* = 0.176, *P*=0.049), log-glucose (*r* = 0.187, *P*=0.036), log-creatinine (*r* = 0.194, *P*=0.029), log-UACR (*r* = 0.198, *P*=0.031), log-insulin (*r* = 0.334, *P* < 0.001), and log-HOMA-IR (*r* = 0.358, *P* < 0.001) positively correlated, whereas HDL-C (*r* = −0.219, *P*=0.014) and eGFR (*r* = −0.325, *P* < 0.001) negatively correlated with serum FGF21 levels in patients with T2DM. Multivariate forward stepwise linear regression analysis of the variables significantly associated with fasting serum FGF21 levels revealed that body fat mass (adjusted *R*^2^ change = 0.218, *P*=0.008) and log-TG (adjusted *R*^2^ change = 0.036, *P* < 0.001) positively correlated, whereas eGFR (adjusted *R*^2^ change = 0.033; *P*=0.013) negatively correlated with serum FGF21 levels in patients with T2DM.

## 4. Discussion

The major findings of our present study are summarized as follows. T2DM patients with MetS have significantly elevated FGF21 concentrations accompanied with a higher proportion of women; higher prevalence of hypertension and elevated BP values; elevated body adiposity items; unfavorable lipid, glucose, and renal function profiles; and increased insulin resistance parameters in comparison with non-MetS individuals with T2DM. FGF21 values have a positive correlation with body fat mass and serum TG level and are negatively correlated with eGFR in T2DM population.

The cluster of interrelated risk factors including hypertension, hyperglycemia, dyslipidemia, and visceral obesity indicates that MetS has cross talk with many cardiometabolic diseases. As expected, our study reveals that T2DM patients with MetS have significantly higher BW, BMI, WC, body fat mass, and SBP and DBP values; higher prevalence of hypertension; elevated TG, fasting glucose, and HbA1c concentrations; and lower HDL-C level than T2DM patients without MetS. Previous epidemiological studies have shown that MetS is closely related to the prevalence of chronic kidney disease (CKD) [18–20]. A systematic review and meta-analysis revealed that MetS and its components have been associated with impaired renal function and microalbuminuria or overt proteinuria [21]. Not surprised, MetS has been associated with increased risks for DM and CVD occurrence, and established cardiovascular risk factors have promoted the development of CKD [22]. Our study also confirmed that significant impaired renal function with elevated UACR and decreased eGFR values is noted in T2DM subjects with MetS than those without MetS.

FGF21, which is primarily secreted by the liver and expressed to a lesser extent in adipocyte, skeletal muscle, pancreas, and thymus, is a hepatokine response to the metabolic imbalance deterioration and has been implicated as a potential biomarker for early detection of these cardiometabolic diseases [23, 24]. FGF21 is initially recognized as a “favorable” cytokine involved in metabolic regulation of insulin-independent glucose transport in cells. An animal study revealed that FGF21 specifically upregulates the glucose transporter 1 (GLUT1) with greater expression of GLUT1 mRNA at the adipocyte cellular membrane and then induces noninsulin-dependent glucose uptake in the insulin resistance model and obesity (ob/ob mice) [6]. Systematically administered FGF21 can lower serum TG and glucose levels and improve lipoprotein profiles significantly in genetically compromised diabetic monkeys [8]. Nevertheless, studies had revealed that serum FGF21 levels are significantly higher in obese patients with MetS components than that in healthy controls, progressively elevated with worsening dysglycemia from normal glucose tolerance to prediabetes and diabetes, and prominently increased in human cardiometabolic diseases such as obesity, MetS, T2DM, coronary artery disease, and nonalcoholic fatty liver disease [9, 10, 23, 25]. A recent study reported that serum FGF21 concentrations were significantly associated with SBP, DBP, BMI, serum TG, and fasting glucose levels in a Japanese adult population without metabolic disorders medication, suggesting that an FGF21 compensatory response to metabolic stress or resistance is associated with “metabolic imbalance” [26].

The mechanism by which FGF21 affects the pathogenesis of the MetS and T2DM is likely to be multifactorial. FGF21 needs to bind to *β*-Klotho complex, an FGF co-receptor expressed prominently in metabolically active tissues such as the liver, adipocyte, and pancreas, to activate FGF receptor–mediated signaling [24]. In the adipocyte, FGF21 promotes white adipose tissue (WAT) uptake of glucose and converts WAT into brown adipose tissue, which also stimulates glucose uptake [27]. In addition, FGF21 can reduce glucolipotoxicity by protecting the pancreas *β*-cells from apoptosis possibly due to its lowering lipid and glucose effects [28]. A previous study suggested that obesity-related adipocyte inflammatory condition can suppress *β*-Klotho expression by tumor necrosis factor-alpha and impair FGF21 function in adipose tissue causing glucose intolerance [29]. Similar actions may also lead to FGF21 resistance in subclinical inflammation such as MetS and T2DM [23]. Accumulating evidence suggests that dyslipidemia has a strong association with inflammatory processes [30, 31]. A study revealed that the circulating FGF21 level has a positive association with high-sensitive C-reactive protein, a parameter of inflammation, in T2DM patients. Besides, high-sensitive C-reactive protein is an independent determinant for the serum FGF21 value in T2DM population [32]. A positive correlation between FGF21 levels and obesity-related parameters such as WC, waist-to-hip ratio, BMI, and body fat percentage were found even after adjusting for age. Furthermore, FGF21 levels progressively elevated when the number of MetS components increased [9].

Our present study revealed that the FGF21 value has correlation with obesity and dyslipidemia parameters such as BMI, WC, body fat mass, and serum TG, HDL-C, and LDL-C levels in the univariate linear regression analysis in T2DM patients and that body fat mass and serum log-TG levels still had positive association with serum FGF21 level even after performing multivariate linear regression analysis, indicating the phenomenon of FGF21 resistance exists in T2DM populations. Tyynismaa et al. reported that liver fat and serum TG levels are the most proximal correlates of circulating FGF21 levels in healthy young adult twins [33]. Novotny et al. have also demonstrated that FGF21 had a positive association with WC and serum TG in MetS population in line with our study [24]. Genetic variations have a moderate role to influence the differences in FGF21 concentrations and may explain the weak relationships between the serum log-TG levels with FGF21 in our study population [33].

Compensatory hyperinsulinemia due to insulin resistance plays a central role in MetS [34]. In line with previous studies, our present study revealed that T2DM patients with MetS have a higher serum insulin level and HOMA-IR value than that of individuals without MetS [14, 34]. Under lipid heparin infusion–induced supraphysiological level of free fatty acid (FFA), the serum FGF21 level was elevated, accompanied with hyperinsulinemia in a recent study. This situation suggested that higher FFA, which is often observed in T2DM populations and presumably secondary to the increased lipolysis, may be one of the main stimulators to increase serum FGF21 in MetS patients with T2DM [35]. As mentioned earlier, high serum FGF21 levels were observed in obesity-related disorders and insulin-resistant patients [9, 10], suggesting FGF21 resistance leading to its compensatory upregulation. This scenario is comparable with hyperinsulinemia and hyperleptinemia in MetS and T2DM [12].

Serum FGF21 levels might also be regulated by renal function. Studies revealed that compared with control volunteers, serum FGF21 levels were eight-fold higher in nondiabetic patients receiving peritoneal dialysis [36]. Median circulating FGF21 values were more than 15-fold higher in hemodialysis patients than in patients with an eGFR >50 mL/min. Furthermore, serum creatinine positively and eGFR negatively predicted FGF21 concentrations in multiple regression analysis in control patients [37]. Our present study, which is also in line with previous studies, revealed that FGF21 value has a negative correlation with eGFR values after multivariate forward stepwise linear regression analysis.

There are some limitations to the current study. First, this was a cross-sectional study with a potential selection bias, and therefore, further longitudinal studies are needed before a cause–effect relationship between serum FGF21 and MetS can be established in the T2DM population. Second, the sample size might have been too small, and some confounders (e.g., smoking) were not included and may have influenced the predictive power in our study [38]. Third, previous study demonstrated that serum FGF21 levels exhibit a major nocturnal rise occurring between midnight and early morning in non-DM patients. Lu et al. also reported that the peak FGF21 levels were observed in the fasting state (8 am.) between participants with and without T2DM [39]. Our present study investigated circulating FGF21 levels only used fasting blood sample as most human studies reported before and may overestimate the serum FGF21 concentrations due to the diurnal rhythm of T2DM patients. Further larger sample studies measuring the 24 hour profile of FGF21 in T2DM individuals are needed to confirm this observation. However, little information is available to determine the interrelationships of FGF21 with the MetS in the T2DM population. This study is the first to examine the relationship between serum FGF21 levels among MetS patients with T2DM. Further animal or clinical studies are needed to determine whether serum FGF21 plays a causal role directly in mediating with MetS in patients with T2DM.

## 5. Conclusions

Serum FGF21, a significant biomarker associated with MetS, levels are positively associated with body fat mass and serum TG level and negatively associated with eGFR values in patients with T2DM.

## Figures and Tables

**Table 1:** Clinical variables of the 126 type 2 diabetes mellitus patients with or without metabolic syndrome.

Variables	All participants (*n* = 126)	No metabolic syndrome (*n* = 42)	Metabolic syndrome (*n* = 84)	*P* value
Age (years)	62.43 ± 12.32	60.83 ± 14.11	63.23 ± 11.32	0.306
Height (cm)	161.48 ± 8.51	162.86 ± 7.70	160.80 ± 8.85	0.201
Body weight (kg)	70.11 ± 13.51	62.94 ± 8.75	73.69 ± 14.07	<0.001^*∗*^
Body mass index (kg/m^2^)	26.77 ± 3.94	23.68 ± 2.45	28.32 ± 3.63	<0.001^*∗*^
Waist circumference (cm)	89.98 ± 9.57	82.25 ± 7.45	93.85 ± 8.07	<0.001^*∗*^
Body fat mass (%)	31.74 ± 7.56	25.48 ± 6.13	34.88 ± 6.13	<0.001^*∗*^
Systolic blood pressure (mmHg)	141.65 ± 20.38	128.76 ± 15.09	148.10 ± 19.66	<0.001^*∗*^
Diastolic blood pressure (mmHg)	82.73 ± 10.92	76.33 ± 8.91	85.93 ± 10.45	<0.001^*∗*^
Total cholesterol (mg/dL)	163.19 ± 30.10	158.40 ± 25.99	165.58 ± 31.83	0.208
Triglyceride (mg/dL)	121.00 (85.00–183.75)	89.50 (58.50–112.25)	136.50 (101.75–217.00)	<0.001^*∗*^
HDL-C (mg/dL)	46.56 ± 12.16	51.10 ± 13.46	44.30 ± 10.83	0.003^*∗*^
LDL-C (mg/dL)	101.18 ± 26.71	96.50 ± 23.50	103.52 ± 28.02	0.165
Fasting glucose (mg/dL)	137.50 (121.00–173.50)	124.00 (115.50–151.75)	142.00 (127.00–182.75)	0.004^*∗*^
Glycated hemoglobin (%)	7.40 (6.60–8.90)	6.90 (6.30–7.80)	7.85 (6.80–9.15)	0.005^*∗*^
Blood urea nitrogen (mg/dL)	16.00 (12.00–18.00)	15.00 (12.00–18.00)	16.00 (12.00–18.75)	0.175
Creatinine (mg/dL)	0. 90 (0.70–1.00)	0.90 (0.70–1.00)	0.90 (0.70–1.00)	0.466
eGFR (mL/min)	85.47 ± 25.64	94.80 ± 28.59	80.80 ± 22.80	0.003^*∗*^
UACR (mg/g)	15.81 (7.15–105.63)	7.95 (4.66–18.33)	25.31 (9.81–190.83)	<0.001^*∗*^
Insulin (*μ*IU/mL)	7.01 (3.25–13.62)	3.65 (1.81–6.17)	9.78 (5.08–18.02)	<0.001^*∗*^
HOMA-IR	2.40 (1.14–4.98)	1.18 (0.77–1.95)	3.63 (1.91–6.77)	<0.001^*∗*^
FGF21 (pg/mL)	192.69 (109.82–283.48)	141.45 (66.16–227.56)	218.95 (139.80–325.63)	<0.001^*∗*^
Women (*n*, %)	47 (37.3)	12 (28.6)	45 (53.6)	0.008^*∗*^
Hypertension (*n*, %)	66 (52.4)	11 (26.2)	55 (65.5)	<0.001^*∗*^
Statin use (*n*, %)	60 (47.6)	15 (35.7)	45 (53.6)	0.058
Fibrate use (*n*, %)	8 (6.3)	1 (2.4)	7 (8.3)	0.196
Metformin use (*n*, %)	69 (54.8)	19 (45.2)	50 (59.5)	0.126
Sulfonylureas use (*n*, %)	70 (55.6)	21 (50.0)	49 (58.3)	0.375
DDP-4 inhibitor use (*n*, %)	77 (61.1)	23 (54.8)	54 (64.3)	0.301
Thiazolidinedione use (*n*, %)	5 (4.0)	2 (4.8)	3 (3.6)	0.747
Insulin use (*n*, %)	30 (23.8)	12 (28.6)	18 (21.4)	0.375

Values for continuous variables are given as means ± standard deviation and are tested by Student's *t*-test. Variables that are not normally distributed are given as medians and interquartile range and are tested by Mann–Whitney *U* test. Values are presented as number (%), and analysis was done using the chi-square test. HDL-C, high-density lipoprotein cholesterol; LDL-C, low-density lipoprotein cholesterol; eGFR, estimated glomerular filtration rate; UACR, urine albumin-to-creatinine ratio; HOMA-IR, homeostasis model assessment of insulin resistance; FGF21, fibroblast growth factor 21; DDP-4, dipeptidyl peptidase 4. ^*∗*^*P* < 0.05 was considered statistically significant.

**Table 2:** Odds ratio for metabolic syndrome by multivariable logistic regression analysis of fibroblast growth factor 21 levels among the 126 patients with type 2 diabetes mellitus.

FGF21 (pg/mL)	Unadjusted		Model 1		Model 2		Model 3	
	OR (95% CI)	*P* value	OR (95% CI)	*P* value	OR (95% CI)	*P* value	OR (95% CI)	*P* value
Per 1 pg/mL	1.008	0.001^*∗*^	1.007	0.004^*∗*^	1.005	0.027^*∗*^	1.005	0.035^*∗*^
FGF21 increase	(1.003–1.012)		(1.002–1.011)		(1.001–1.010)		(1.000–1.010)	

Model 1 is adjusted for age and gender. Model 2 is adjusted for the Model 1 variables and for estimated glomerular filtration rate and urine albumin-to-creatinine ratio. Model 3 is adjusted for the Model 2 variables and for insulin level and homeostasis model assessment of insulin resistance. ^*∗*^*P* < 0.05 by multivariate logistic regression analysis. FGF21, fibroblast growth factor 21; OR, odds ratio; CI, confidence interval.

**Table 3:** Correlation between serum fibroblast growth factor 21 levels and clinical variables among the 126 patients with type 2 diabetes mellitus.

Variables	Logarithmically transformed fibroblast growth factor 21 (pg/mL)				
	Univariate			Multivariate	
	*R*	*P* value	Beta	Adjusted *R*^2^change	*P* value
Women	0.172	0.055	—	—	—
Hypertension	0.201	0.024^*∗*^	—	—	—
Age (years)	0.121	0.179	—	—	—
Height (cm)	–0.059	0.513	—	—	—
Body weight (kg)	0.168	0.060	—	—	—
Body mass index (kg/m^2^)	0.259	0.003^*∗*^	—	—	—
Waist circumference (cm)	0.301	0.001^*∗*^	—	—	—
Body fat mass (%)	0.359	<0.001^*∗*^	0.218	0.218	0.008^*∗*^
SBP (mmHg)	0.191	0.032^*∗*^	—	—	—
DBP (mmHg)	0.180	0.043^*∗*^	—	—	—
Total cholesterol (mg/dL)	0.163	0.068	—	—	—
Log-triglyceride (mg/dL)	0.499	<0.001^*∗*^	0.357	0.036	<0.001^*∗*^
HDL-C (mg/dL)	–0.219	0.014^*∗*^	—	—	—
LDL-C (mg/dL)	0.176	0.049^*∗*^	—	—	—
Log-glucose (mg/dL)	0.187	0.036^*∗*^	—	—	—
Log-HbA1c (%)	0.059	0.517	—	—	—
Log-BUN (mg/dL)	0.001	0.996	—	—	—
Log-creatinine (mg/dL)	0.194	0.029^*∗*^	—	—	—
eGFR (mL/min)	–0.325	<0.001^*∗*^	–0.205	0.033	0.013^*∗*^
Log-UACR (mg/g)	0.198	0.031^*∗*^	—	—	—
Log-insulin (*μ*IU/mL)	0.334	<0.001^*∗*^	—	—	—
Log-HOMA-IR	0.358	<0.001^*∗*^	—	—	—

Data of triglyceride, glucose, HbA1c, BUN, creatinine, UACR, insulin, and HOMA-IR levels showed skewed distribution and therefore were log-transformed before analysis. Analysis of data was done using the univariate linear regression analysis or multivariate stepwise linear regression analysis (adapted factors were hypertension, body mass index, waist circumference, body fat mass, SBP, DBP, log-triglyceride, HDL-C, LDL-C, log-glucose, log-creatinine, eGFR, log-UACR, insulin, and HOMA-IR). SBP, systolic blood pressure; DBP, diastolic blood pressure; HDL-C, high-density lipoprotein cholesterol; LDL-C, low-density lipoprotein cholesterol; HbA1c, glycated hemoglobin; BUN, blood urea nitrogen; eGFR, estimated glomerular filtration rate; UACR, urine albumin-to-creatinine ratio; HOMA-IR, homeostasis model assessment of insulin resistance. ^*∗*^*P* < 0.05 was considered statistically significant.

## Data Availability

The data used to support the findings of this study are included within the article.
